# Do Illegitimate Tasks Lead to Work Withdrawal Behavior among Generation Z Employees in China? The Role of Perceived Insider Status and Overqualification

**DOI:** 10.3390/bs13090702

**Published:** 2023-08-23

**Authors:** Pengxiang Fan, Hao Zhang, Songlin Yang, Zixuan Yu, Ming Guo

**Affiliations:** 1 School of Economics and Management, Beijing Jiaotong University, Beijing 100089, China; 2 School of Economics, University of Amsterdam, 1018 WV Amsterdam, The Netherlands

**Keywords:** illegitimate task, work withdrawal behavior, perceived insider status, perceived overqualification, Generation Z employee

## Abstract

Generation Z employees in the workplace cause a management challenge that enterprises have recently faced. The unique characteristics of Generation Z employees necessitate an urgent update to the knowledge of organizational management. However, few studies of the literature focus on the workplace behaviors of Generation Z. This study proposes that illegitimate tasks may lead to work withdrawal behavior among Generation Z employees. Based on the equity theory model, this study constructed a moderated mediation model to explore the impact of illegitimate tasks on the work withdrawal behavior of Generation Z employees, as well as the mediating role of perceived insider status and the moderating role of perceived overqualification. The analysis of survey data from 283 Generation Z employees in China at two time points found that illegitimate tasks are positively correlated with work withdrawal behavior. At the same time, the mediating role of perceived insider status was successfully confirmed. The results also showed that perceived overqualification strengthened the effect of illegitimate tasks on work withdrawal behavior and the mediating effect of perceived insider status. This study offers new insights into the management and development of Generation Z employees and the sustainable evolution of workplace relationships from both theoretical and practical perspectives.

## 1. Introduction

Most scholars define Generation Z (Gen Z) as those born between 1995 and 2010 [1,2] since the maximum span of a generation should be 15 years [3]. The Gen Z employees focused on in this study refer to those in the workforce who have reached the legal working age, thus their age range is more specific and narrower. As the newest and youngest cohort entering the global workplace, Gen Z employees have become a growing concern for leaders worldwide, surpassing Generation Y in size [4]. Approximately 170 million Gen Z employees in China exist or are preparing to join the workforce. Gen Z employees display distinct traits compared to previous generations [5,6]. They are known for their values of diversity and inclusion, as well as their desire for transparency and authenticity in their work environment. Meanwhile, the characteristics of Gen Z can be influenced by culture and socio-economic conditions. Generation Z in China was born and raised against the backdrop of rapid economic growth. They have lived through many significant historical events, such as the comprehensive establishment of a market economy system in 1992, the introduction of the Internet in 1994, the full integration of China into globalization in 2003, and the Beijing Olympics in 2008. Due to being in a period of rapid social development, Chinese Generation Z employees exhibit complex characteristics. To be specific, as the first “global generation” [7], Chinese Gen Z shares traits like being “digital natives” with their global counterparts. The advent and widespread use of digital communication technologies have given them increased access to the Internet, mobile phones, and social media, molding their distinct expression modes and value systems. Also, China’s Gen Z lives in a relatively affluent social stage, where they mainly pursue self-value, personal happiness, individualization, openness, and fairness at the psychological level. Moreover, China’s Gen Z has generally received a good education. Therefore, Gen Z employees in China will demonstrate traits of individualism, readiness to challenge leadership, strong self-esteem, and pursuit of fairness [8,9,10] in the workplace. Faced with similar situations as other generations, they may respond differently. As Gen Z’s presence in the workplace expands, the need for organizations to comprehend their unique attitudes and behaviors should be highlighted. Mitigating negativity and ensuring better progression for this newest generational cohort is crucial.

Work withdrawal behavior has recently become increasingly important among China’s Gen Z employees. Topics related to work withdrawal behavior, such as “lying flat” (a trend indicating passive resistance against work), have become phenomenally popular among young people on social media [11]. The search index for these topics (69,162) surpassed COVID-19 (11,741) on the most prominent Chinese search engine. Work withdrawal behavior is defined as actions by organizational members to avoid their job roles and tasks while maintaining organizational functionality and job role relationships, such as deliberately not doing work within their duties [12]. Work withdrawal behavior is categorized into psychological withdrawal and behavioral withdrawal. Typical work withdrawal behavior includes daydreaming, leaving a workstation for unnecessary reasons, leaving early, and putting less effort into the job than they should have. Work withdrawal behavior has negative effects. The work withdrawal behavior of Gen Z employees will not only affect individual work performance [13], but also will inevitably affect corporate performance [14] and management costs [15]. While numerous studies have analyzed the emergence of work withdrawal behavior trends from macro perspectives, including COVID-19 and economic recession, micro factors within the workplace have yet to be observed. Thus, it becomes a significant matter for academia and industry to examine the causes of work withdrawal behavior among Generation Z employees from the workplace perspective to implement preventive measures.

In professional workplace settings, employees often find themselves dealing with tasks incongruent with their reasonable duties and expectations, such as completing leaders’ personal chores or being forced to attend business banquets. These tasks are categorized as ‘illegitimate tasks’ in the study of organizational behavior [16]. ‘Illegitimate tasks’ refers to when work employees seem to violate reasonable norms or expectations associated with their roles [17], subdivided into ‘unreasonable’ and ‘unnecessary’ tasks. The former encompasses tasks deemed to exceed employees’ role scope or misalign with their expertise, like assigning administrative duties to professional teachers. The latter includes tasks perceived as pointless, leading to unnecessary effort, wasted time, and organizational inefficiencies, such as sorting through worthless data [16]. Illegitimate tasks violate employees’ subjective norms and expectations rather than objective organizational regulations. When employees perceive tasks as unreasonable or unnecessary, they are deemed illegitimate tasks, even if they may not be so objectively. In the United States, illegitimate tasks constitute a third of employees’ daily workload [16]. However, this phenomenon is even more prevalent in high power distance environments like Chinese workplaces [18]. Gen Z employees in China, more independently self-directed compared to older generations, may perceive and identify more tasks as illegitimate. As a more vulnerable group within organizations, these younger employees are often delegated such tasks [19]. However, past studies have focused on the organizational, leadership, and individual factors influencing work withdrawal behavior [20,21,22] but neglected the role of work tasks. Thus, empirical research investigating whether illegitimate tasks incite work withdrawal behavior among Gen Z employees is essential.

This research aims to understand the potential of illegitimate tasks to trigger work withdrawal behavior. Illegitimate tasks have previously been associated with negative workplace behavior, such as counterproductive work behavior [23,24]. When faced with such tasks, Gen Z employees in lower positions may resort to covert retaliation [25]. Also, Gen Z employees rarely engage in direct organizational harm through other dimensions of counterproductive behaviors such as sabotage or theft [26]. Therefore, as covert and mildly counterproductive behavior, work withdrawal behavior could be tied to illegitimate tasks. While previous research indicates illegitimate tasks can lead to counterproductive behavior, the precise relationship between illegitimate tasks and the dimensions of counterproductive behavior remains largely unexplored, leading to potential oversimplification of the impact of illegitimate tasks on specific employee groups. By examining the effect of illegitimate tasks on Gen Z’s work withdrawal behavior, we can better understand the specific range of these tasks’ negative impact on Gen Z, providing targeted insights for workplace behavior management. Given the potential value in reducing Gen Z’s work withdrawal behavior through improved task assignments, investigating the relationship between illegitimate tasks and work withdrawal behavior is crucial.

This study proposes a cognitive mechanism based on equity theory to elucidate the relationship between illegitimate tasks and the work withdrawal behavior of Generation Z employees. The equity theory suggests that unfair work events can trigger negative cognitive responses in individuals, affecting their work behavior [23]. Based on equity theory, this study aims to explain the perspective of perceived insider status. Perceived insider status is an individual’s understanding of the personal space, status, and acceptance they receive within a specific organization as a member of said organization [26]. For example, people feel very much a part of their work organization or believe that they are included in the organization. However, illegitimate tasks make employees feel that they are not a part of the organization because, compared to others within the organization, they perceive unfair treatment from the organization. Illegitimate tasks are instances of workplace injustice, by disrupting the employees’ perception of the organization as fair, and illegitimate tasks signal to the employees that they are not accepted or respected by the organization, which may trigger work withdrawal behavior. Previous studies have shown that perceived insider status can enhance positive work behaviors, such as constructive work behavior [27], and reduce negative work behaviors, like an individual’s intention to leave and increase staff turnover [28]. Furthermore, shaped by the traditional culture of collectivism [29], individuals in China tend to emphasize circle-based psychological feelings during workplace interactions. Primarily due to the former one-child policy in China, individuals have been raised with familial attention and care [30], therefore Generation Z employees in China strongly need to be accepted and respected by their organization, leaders, and colleagues, and feel a sense of belonging in the workplace. When Generation Z employees perceive unfairness and fail to satisfy their need for organizational belonging, they may resort to work withdrawal behavior. Therefore, we will focus on the mediating role of perceived insider status between illegitimate tasks and withdrawal behavior.

This study tries to examine the conditions under which illegitimate tasks might trigger work withdrawal behavior among Generation Z employees. We introduce “overqualification”, a significant personal attribute that impacts task perceptions, as a moderating factor. Overqualification refers to an individual’s perception of education, skills, or capabilities surpassing their current job requirements [31], which can be divided into objective overqualification and subjective overqualification. Since subjective measurements are the most efficient way to study overqualification and subjective perceptions typically predict work attitudes and behaviors more effectively than objective circumstances, subjective overqualification will be recognized in this study. Subjective overqualification is known as perceived overqualification (POQ) in organizational behavior research. The items on the perceived overqualification scale include “My job requires less education than I have” and “I have more abilities than I need in order to do my job”. The issue of overqualification in Chinese workplaces became a subject of academic interest as early as 2015 [32]. The expansion of higher education and increasing market competition have recently escalated perceived overqualification. The studies indicate that 84% of employees in China feel that their qualifications exceed job requirements [18], a sentiment that is prevalent among China’s Generation Z employees [33,34], as evidenced by individuals who possess PhDs from top universities like Tsinghua serving in community roles instead of research [35]. This study posits that employees expect their superior skills and knowledge to receive commensurate fairness from their leaders. Unmet expectations can instigate a sense of unfairness, leading to more negative cognitions and, subsequently, more negative workplace behaviors [36,37]. In highly overqualified employees, tasks may be seen as illegitimate much more easily, exacerbating negative attitudes and unfairness. This can harm their perceived insider status and strengthen the negative relationship between illegitimate tasks and withdrawal behavior. Therefore, we further discuss the moderating mechanism of perceived overqualification between perceived insider identity and work withdrawal behavior.

**Figure 1:**
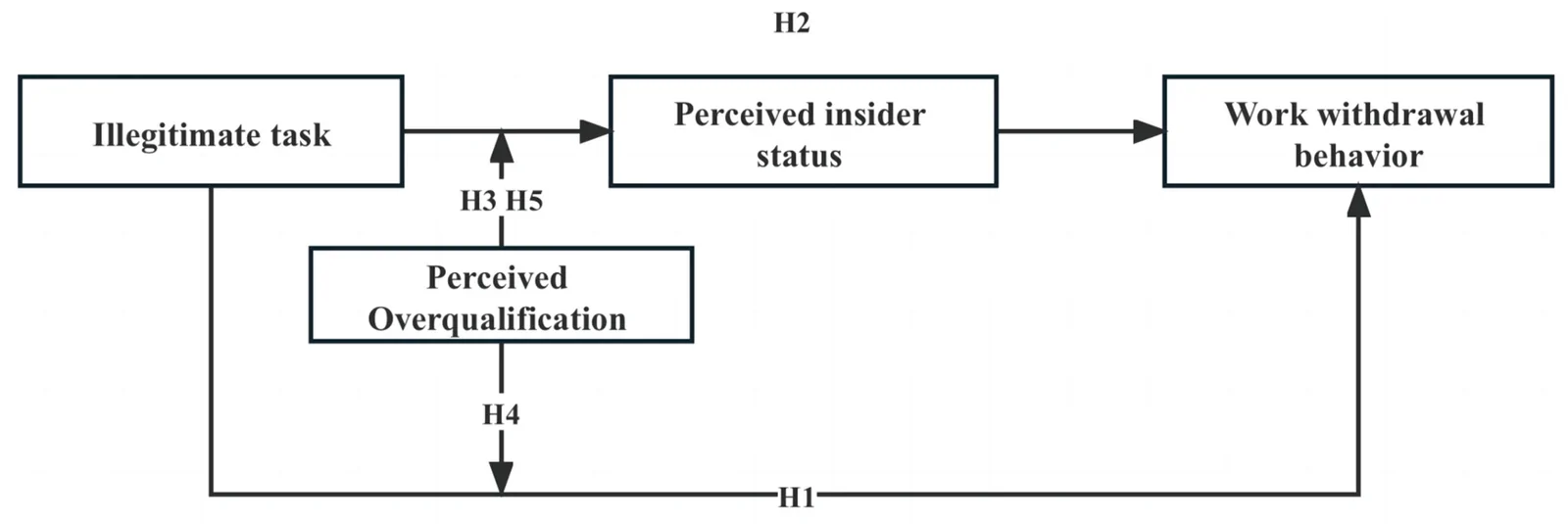
Theoretical model.

To summarize, this study aims to explore the impact of illegitimate tasks on the work withdrawal behavior of Generation Z employees. Specifically, it seeks to elucidate the psychological mechanism of the influence of illegitimate tasks on employees’ work withdrawal behavior through the lens of perceived insider status. Furthermore, this research unveils the role of perceived overqualification as a boundary condition, illustrating the situational factors of the negative effects of illegitimate tasks. This study proposes a moderated mediation model, as shown in Figure 1.

Our research contributes in the following significant ways to the existing literature: Firstly, this study examines task-related factors on employees’ work withdrawal behavior. Prior studies have primarily focused on the organization [20], leadership [21], and individual attributes [22], neglecting the influence of work tasks. While it is established that illegitimate tasks can harm employees, understanding is limited on how employees might retaliate covertly. Our study, thus, deepens insight into the impact of illegitimate tasks on withdrawal behavior. Secondly, our research extends knowledge of the cognitive mechanisms underpinning the impact of illegitimate tasks on withdrawal behavior. Grounded in equity theory, we propose that illegitimate tasks disrupt individuals’ perception of fairness-based status. Our work augments the existing literature by introducing perceived insider status as a novel mediating factor. Besides, we investigate the previously overlooked moderating role of qualification factors [38], providing a more comprehensive viewpoint. Finally, by focusing on Generation Z employees, our research diversifies the investigation into the relationship between illegitimate tasks and work withdrawal behavior. Despite substantial research on the negative consequences of illegitimate tasks [39], studies targeting specific demographics like Gen Z are scarce. We address this gap, scrutinizing Gen Z’s attitudes and responses to unavoidable illegitimate tasks. Additionally, we account for non-Western contexts’ cultural and social nuances, often overlooked in the existing literature [40], thus providing a more accurate portrayal of Gen Z’s behavior globally. Lastly, unlike most studies confined to the hotel and tourism industries [41,42], we consider Gen Z employees across diverse industries, enhancing our findings’ applicability and practical value.

## 2. Theoretical Background and Hypotheses

### 2.1. Theoretical Background

According to prior investigations into equity theory [43,44,45], two kinds of individual responses exist within professional environments. First, how individuals perceive and assess organizational fairness significantly affects their cognitive processes, attitudes, and behaviors at work [46]. Those who perceive themselves as receiving inequitable treatment may attempt to restore balance [24]. Organizational fairness encompasses distributive fairness, procedural fairness, and interactional fairness [47]. When employees perceive themselves as engaging in illegitimate tasks, they not only regard these tasks as a result of unfair distribution [48] but also perceive them as demonstrations of procedural and interactional unfairness because such tasks disrupt professional roles, interpersonal relationships, and self-esteem [16,49]. As a result, these individuals perceive illegitimate tasks as organizational injustices, inciting negative cognitions and behaviors [23].

Moreover, there is a distinct aspect within equity theory. The theory posits that employees usually measure their input–output ratios against those of their colleagues. If they perceive their input–output ratio to be lower than others, they sense unfairness, culminating in negative attitudes and actions aimed at recapturing fairness [45,50]. Specifically, when individuals perceive overqualified status— discerning a discrepancy between their assigned tasks and their education, experience, and skills, and when they fail to gain particular value from these tasks— they perceive a lower input-output ratio. Consequently, these perceive overqualified employees experience unfairness, reinforcing negative attitudes and stimulating changes in their input or output to mitigate the sense of inequity [37].

In conclusion, when employees are faced with illegitimate tasks, they may perceive these tasks as manifestations of distributive injustice. This is because they are treated unfairly by the organization and also do not receive a fair return. They may also feel disrespected, and if the decision to assign these tasks was made unfairly, this threatens their social esteem and professional identity. In this context, illegitimate tasks represent procedural and interactional injustices. Secondly, due to the unfair characteristics of illegitimate tasks, employees will attribute these tasks to the organization, leading them to feel unaccepted. Research has shown that illegitimate tasks can reduce employees’ perception of organizational fairness, leading to negative cognition and behavior. Lastly, as a result of illegitimate tasks, employees may feel an effort–reward imbalance. In such cases, individuals will try to restore balance by changing their work attitudes and behavior, that is, reducing effort or maximizing reward, thus producing negative responses.

### 2.2. Illegitimate Task and Work Withdrawal Behavior

Illegitimate tasks, defined as tasks that breach the reasonable expectations and norms associated with individuals, can be divided into two dimensions: unnecessary tasks and unreasonable tasks [16,51]. These tasks are hallmarked by their violation of role boundaries and infringement on individuals’ professional identity. This study argues that illegitimate tasks embody unfair attributes within the organization, specifically concerning their disruption to the three dimensions of organizational fairness. Firstly, distributive fairness in the workplace primarily concerns how individuals perceive the fairness of resource allocation [52]. When individuals perceive illegitimate tasks as specific outcomes directed at themselves, they view these tasks as unnecessary compared to the standard tasks carried out by their colleagues or within their own job responsibilities [53]. Furthermore, illegitimate tasks, as they are informal tasks outside individuals’ occupational roles, fail to offer expected compliance-related benefits [54]. Therefore, employees perceive the assignment of illegitimate tasks as an unfair distribution. Secondly, procedural fairness emphasizes the fairness of the decision-making processes [55]. The allocation process of illegitimate tasks lacks consistency among organizational members, with these tasks being assigned only to a subset of employees. As passive recipients of task assignments, employees are excluded from the decision-making process concerning illegitimate task allocation. Moreover, illegitimate tasks include unreasonable tasks that contradict the fairness of organizational systems. Therefore, individuals may perceive the procedures for assigning illegitimate tasks as unfair. Thirdly, interactional fairness deals with the impact of interpersonal interactions on the sense of fairness, which includes both interpersonal and informational fairness [56]. Work tasks act as social information about employees. Existing research indicates that illegitimate tasks offend individuals’ professional identity and erode their self-esteem [17,24], making employees feel disrespected during interactions with their superiors. Under such circumstances, employees perceive themselves as being treated unfairly.

According to equity theory, when employees perceive unfairness, they attempt to counteract the adverse effects of unfairness by altering their attitudes and behaviors at work. Withdrawal behaviors refer to a series of intentional attitudes or actions employed by employees to avoid work, distance themselves from the organization, or weaken their connection with the organization when they perceive displeasing circumstances [57]. In societies with a high power distance, responses to illegitimate tasks are more likely to be manifested through covert and safe destructive behaviors. When individuals perceive themselves as being treated unjustly by the organization, they are likely to engage in varying degrees of passive work resistance behavior to balance their input and rewards, and eliminate or reduce the experience of unfairness [25]. Moreover, due to the inherent unfairness, illegitimate tasks can provoke negative behaviors in individuals [58,59]. Substantial empirical research shows that illegitimate tasks can lead to counterproductive behaviors [24,60]. Conversely, when employees do not encounter illegitimate tasks, they perceive a higher level of organizational fairness and demonstrate more extra-role behaviors [61]. Based on these insights, this study proposes the following hypothesis:

**Hypothesis** **1.** *Illegitimate tasks have a significant positive influence on work withdrawal behavior among Generation Z employees*.

### 2.3. The Mediating Role of Perceived Insider Status

Equity theory posits that employees are attuned to fairness in the workplace [62]. When individuals encounter an unfair environment due to being assigned tasks that violate organizational norms, their positive cognitive processes are adversely affected. The perception of insider status is grounded in the way employees perceive differential treatment within the organization, their experience of self-relevance and value, and the degree to which they are regarded as “insiders” by the organization [63]. Illegitimate tasks, defined as unfair events within the organization, reflect the unfairness of task assignments to employees, potentially diminishing the impact of employees’ perception of insider status. The following rationale can explain this: From the direct perspective of employee–leader interaction, illegitimate tasks convey social information signifying disrespect, lack of appreciation, and harm to the individual’s self-esteem. This implies a low employee status within the organization, making it difficult for the individual to perceive appreciation from the organization [64,65]. Consequently, this increases self-doubt and a sense of unfairness. From the indirect perspective of employee perception of the organization, employees who encounter illegitimate tasks experience a discrepancy between the tasks and the organizational systems and goals. Illegitimate tasks are inconsistent with their daily job responsibilities and threaten their professional roles [66]. As a result, employees who encounter illegitimate tasks perceive a need for more consistency between the tasks and the organizational system and goals, leading them to believe they are being treated differently by the organization. In such a situation, employees perceive illegitimate tasks as unfair treatment, thereby inhibiting the evolution of their insider status perception.

In fact, on the one hand, illegitimate tasks are perceived as unfair and can diminish individuals’ level of perceiving themselves as “insiders”. On the other hand, employees who perceive unfairness actively undermine their perceiving insider status in an attempt to mitigate the negative feelings associated with unfairness and regain a sense of fairness. In particular, Generation Z employees, who have been doted on by their families since childhood, seek recognition and affirmation from leaders and organizations in the workplace. Illegitimate tasks, which are objective breaches of fairness, fail to signal to employees that they are integral to the organization, thereby obstructing the enhancement of insider identity status among Generation Z employees. Therefore, illegitimate tasks negatively affect the insider identity perception of Generation Z employees.

Furthermore, perceived insider status broadcasts to employees that they are a vital part of the organization, reflecting their status or identity. Employees’ perception of fairness is a crucial factor influencing their withdrawal behavior [67]. Research has revealed that the perception of insider identity can stimulate positive behaviors such as voicing opinions, innovative behavior, and job engagement among employees [68,69,70]. It has significantly negated employees’ deviant behavior and turnover [63,71]. Behavior is shaped by cognition and attitudes, especially when individuals perceive organizational unfairness and a sense of not being accepted within the “group”. Under such circumstances, employees may engage in work withdrawal behavior to restore fairness between themselves and the organization. When employees struggle to perceive themselves as internal members of the organization, fostering a robust sense of belonging and identification with the organization becomes challenging. This perceived lack of status makes it difficult for employees to establish a solid emotional and behavioral connection with the organization, leading to work withdrawal behavior. On the other hand, when employees perceive themselves as internal members, they often experience a stronger sense of belonging and identification with the organization. This makes them more inclined to engage in organizational citizenship behaviors and innovative behaviors [72]. They proactively generate more positive work behaviors to bolster their connection with the organization.

Compared to previous generations, Generation Z employees have been raised during rapid economic, medical, and educational advancements. Their basic physiological and safety needs are generally met, but they have a heightened need for emotional belonging and respect. A diminished sense of internal identity fails to satisfy their social and emotional needs. As a result, Generation Z employees are prone to adopt a more hostile work attitude, leading to increased work withdrawal behavior.

To summarize, perceived insider status can positively influence employees’ work withdrawal behavior. Perceived insider status is an important mediating factor between illegitimate tasks and work withdrawal behavior. Illegitimate tasks convey signals of organizational unfairness, disrespect, and lack of recognition to employees, negatively impacting their perception of internal identity and increasing work withdrawal behavior. Illegitimate tasks represent manifestations of unfairness and bear characteristics of unfair treatment. They heighten employees’ perception of being treated differently. In response to this organizational antagonism and retaliation, employees may curtail their work efforts within their job responsibilities and engage in covert work withdrawal behavior. Based on this, hypothesis 2 is proposed in this study:

**Hypothesis** **2.** *Perceived insider status mediates the relationship between illegitimate tasks and work withdrawal behavior*.

### 2.4. Moderating Effect of Perceived Overqualification

The intensity of the relationship between illegitimate tasks, perceived insider status, and employees’ work withdrawal behavior may vary among employees with different levels of perceived overqualification. Equity theory posits that employees usually compare their input–output ratio with other employees. When they sense their input–output ratio to be lower than others, they feel unfairly treated, leading to negative attitudes and engagement in negative behaviors. Perceived overqualification implies that employees invest more in education, knowledge, skills, and experience than the outcomes they receive regarding opportunities and status [73].

Highly perceived overqualified employees tend to have lower positive perceptions of the organization, more insufficient recognition of the value of their work, and higher levels of negative self-perceptions. As their abilities exceed the job requirements, they may feel that the organization must fully recognize them. Specifically, firstly, employees with high-perceived overqualification are more confident in their abilities and experience a sense of superiority. They always expect the organization to provide matching jobs and preferential treatment. As a result, when they encounter illegitimate tasks, highly perceived overqualified employees will find it challenging to meet their expectations, leading them to become more frustrated and angry with the organization [74]. Moreover, highly perceived overqualified employees are more sensitive to unfairness. When their investments in knowledge and skills do not find opportunities for application, they amplify the gap between their expectations and reality, intensifying their sense of unfairness. Under conditions of a low sense of fairness, employees’ perception of insider status is weakened, and they are more likely to perceive themselves as outsiders of the organization. Existing research has demonstrated that highly perceived overqualified employees engage in more job search behaviors, voluntary turnover, and counterproductive work behaviors [75,76,77].

Generation Z employees, on the one hand, are mainly the only children in their families due to China’s family planning policy, which results in receiving more attention and investment in education from their families. In addition, influenced by the expansion of higher education enrollment, they have more opportunities to enter universities and pursue further studies. Consequently, Generation Z employees generally possess higher educational qualifications, knowledge, and skills. On the other hand, the widespread availability of higher education has led to challenges in employment for Generation Z, forcing them to lower their job expectations and accept positions that may be below their education and skill levels, which creates a subjective perception of overqualification. Furthermore, when analyzing the personality traits of Generation Z employees, they tend to be more assertive, self-centered, and have a strong sense of self-efficacy. They often underestimate the importance of job tasks and overestimate their abilities, reinforcing the overqualification perception. Based on the above arguments, this study proposes Hypothesis 3 and Hypothesis 4:

**Hypothesis** **3.** *Perceived overqualification has a positive moderating effect on the relationship between illegitimate tasks and perceived insider status*.

**Hypothesis** **4.** *Perceived overqualification has a positive moderating effect on the relationship between illegitimate tasks and work withdrawal behavior among Generation Z employees*.

High-perceived overqualification employees tend to have a more negative attitude towards illegitimate tasks, as they perceive such unfair events and lack the motivation to further integrate into the “circle”. They exhibit lower enthusiasm for related work and reduce their investment in order to seek fairness. On the contrary, employees with a common sense of overqualification need more fair experience. They are more challenging in their identification with illegitimate tasks and have a higher tolerance for, and think that they can play, their due role in the organization, making it easier to show commitment to work. Therefore, based on the theoretical derivation from Hypothesis 1 to Hypothesis 4, this study suggests that perceived overqualification not only moderates the relationship between illegitimate tasks and perceived insider status but also moderates the mediating effect of perceived insider status on the relationship between illegitimate tasks and work withdrawal behavior among Generation Z employees. Based on this, Hypothesis 5 is proposed:

**Hypothesis** **5.** *Perceived overqualification positively moderates the mediating effect of perceived insider status on the relationship between illegitimate tasks and work withdrawal behavior*.

## 3. Method

### 3.1. Samples and Procedure

We collaborated with part-time MBA students in professional courses. These MBA students come from 17 companies in the transportation, digital technology, trade, software, and finance industries, of which 80% are state-owned or private medium and large enterprises, with an average number of employees of approximately 300. We chose companies from these industries because they have a higher number of young people with advanced education compared to traditional manufacturing industries. Most MBA students serve as leaders or ordinary employees in the human resources department and mainly engage in related human resource management work, such as communication about compensation. We selected our sample according to the following procedure: First, they helped us recruit participants from the companies, and a recruitment email was sent to potential participants to introduce the purpose and procedure of the study. Then, those employees who were interested and qualified would provide us with their contact information and email. Participants were selected using the simple random sampling method. Finally, according to the previous literature [78], we distributed questionnaires to participants through an online questionnaire platform similar to MTurk, Credamo, and emails. We numbered each survey questionnaire with employee IDs to ensure data matching. We also assured each participant that their data would be kept confidential and would only be used for academic research.

We collected data in two stages with an interval of one month. Previous research has used a one-month lag to collect two waves of data to understand the relationship between illegitimate tasks and behaviors [79,80]. Therefore, one month is enough to separate the measurement time points of independent and dependent variables to avoid common method bias and confusion of causal relationships. On 16 December 2022, we distributed 350 questionnaires to collect demographic information, illegitimate tasks, and perceived overqualification. On 15 January 2023, we sent questionnaires to the employees who participated in the first stage of the survey to collect data on perceived insider status and work withdrawal behavior. Based on the age standard of Generation Z (1995–2010) and the shortest time for answering the questionnaire (>180 s) [81], invalid questionnaires were excluded. Finally, we obtained 283 valid samples. According to common research [82], we use the ratio of the number of received valid questionnaires to the total number of distributed questionnaires as the response rate. The sample response rate of this study is 80.86%. The response rate is an indicator of sample representativeness and data quality. Therefore, the response rate of this study indicates a high level of participant involvement in the research, meaning that most of the data can be used for further analysis and research, making the research results more representative and universal.

The valid sample for this study consisted of 43.0% male employees and 57.0% female employees; the average age was 24.93 years (SD = 2.06); 4.8% had been with their current company for less than 1 year, 29.6% for 1–2 years, 43.0% for 3–4 years, and 22.6% for more than 5 years; the educational level was 12.6% for college and below, 80.4% for bachelor’s degree, and 7.0% for master’s degree and above; 74.4% for finance, 10.4% for digital technology, 1.5% for trade, 6.7% for transportation, and 7% for software; 35.2% worked in state-owned enterprises, 53.0% in private enterprises, 9.6% in foreign-funded/joint venture enterprises, and 2.2% in others.

### 3.2. Measures

In this study, we used established scales for variable measurement. The scales were professionally translated and back-translated to ensure semantic accuracy, and the scoring method was based on the Likert 5-point scale. We have provided a detailed presentation of the variable scales (see Appendix A).

Illegitimate Task. We used the Illegitimate Task Scale developed by Semmer et al. [83]. This scale includes two dimensions: unnecessary task and unreasonable task, with eight questions. Sample items included “I get tasks I don’t see any point in doing” and “I have work tasks to take care of, which keep me wondering if they even have to be done at all”. The Cronbach’s alpha coefficient is 0.920.

Perceived Overqualification. We used the 9-item scale of Maynard et al. [31] to measure perceived overqualification. Sample items included “My job requires less education than I have” and “The work experience that I have is not necessary to be successful on this job”. The Cronbach’s alpha coefficient is 0.968.

Perceived Insider Status. The perceived insider status is measured based on the scale developed by Stamper and Masterson [63], which includes six questions. Sample items included “I feel very much a part of my work organization” and “I feel like I am an ‘outsider’ at this organization”. The Cronbach’s alpha coefficient is 0.940.

Work Withdrawal Behavior. We used the scale developed by Lehman and Simpson [84] to measure work withdrawal behavior. This scale includes a total of 12 questions. Sample items included “Thoughts of being absent” and “Left work station for unnecessary reasons”. The Cronbach’s alpha coefficient is 0.976.

Control Variables. Considering that employees’ positions, education, gender, and age can impact their reactions to job stressors and their behavior [85], we used employees’ gender, age, education, years of experience, company category [66], and industry as control variables (male = 1, female = 0; college or less = 1, bachelor’s degree = 2, master’s degree or above = 3; less than 1 year = 1, 2–3 years = 2, 4–5 years = 3, more than 5 years = 4; state-owned enterprise = 1, private enterprise = 2, joint venture/foreign investment enterprise = 3, other = 4; finance = 1, digital technology = 2, trade = 3, transportation = 4, software = 5).

### 3.3. Statistical Analysis Methods

This study used Amos 22.0 for confirmatory factor analysis of the data, SPSS 23.0 for reliability testing, common method bias, descriptive statistics, correlation analysis, mediation effect analysis, and moderated effect testing. We also used the PROCESS plug-in to further validate the mediation effect and mediated effect testing with moderation.

## 4. Result

### 4.1. Confirmatory Factor Analysis

The results of the confirmatory factor analysis are shown in Table 1. Compared to other factor models, the four-factor model presented the best-fit indices (χ^2^/df = 2.199, RMSEA = 0.067, CFI = 0.932, TLI = 0.925), and the factor loadings for all factors were significant. This indicates that the four variables involved in this study exhibited good discriminant validity.

Furthermore, this study used Harman’s single factor test to examine the potential issue of common method bias. The results showed that the first factor explained 23.91% of the variance, below the critical value of 40%, preliminarily suggesting no severe common method bias. To control for the potential underestimation of common method bias by Harman’s single-factor test, we further employed an unmeasurable latent method factor control method for testing [86]; that is, a dual-factor model was established by adding a method factor as a global factor on the basis of the originally designed factors. If the fit indices of the original four-factor CFA model significantly differ after adding the latent method factor, this suggests a serious common method bias [87]. As shown in Table 1, the fit indices of the model did not significantly improve after adding the common method factor to the four factors (changes in RMSEA, SRMR, CFI, and TLI did not exceed 0.01), further validating that there was no serious common method bias in this study.

### 4.2. Descriptive Statistical Analysis

Table 2 presents the means, standard deviations, and correlation coefficients of the study variables. The results show that illegitimate tasks were significantly and negatively correlated with perceived insider status (r = −0.330, *p* < 0.01) and significantly and positively correlated with work withdrawal behavior (r = 0.325, *p* < 0.01). Perceived insider status was significantly and negatively correlated with work withdrawal behavior (r = −0.463, *p* < 0.01). These correlations align with the theoretical model and provide preliminary support for the hypothesis testing.

### 4.3. Hypothesis Testing

#### 4.3.1. Main Effect and Moderating Effect Tests

The following employs the least squares method for regression analysis, testing both main and moderating effects. The results of the regression analysis are presented in Table 3. All models have been controlled for employee gender, age, years of work experience, education, industry category, and enterprise type.

In Model 1, only the control variables and illegitimate task were included in the regression. The results showed a significant negative association between illegitimate task and perceived insider status (Model 1, β = −0.322, *p* < 0.001).

Model 2, based on Model 1, added perceived overqualification and the interaction term between illegitimate task and perceived overqualification. Illegitimate task remained negatively associated with perceived insider status (β = −0.289, *p* < 0.001). The interaction between illegitimate task and perceived overqualification was negative and significant (β = −0.141, *p* < 0.01), indicating that perceived overqualification strengthens the negative relationship between illegitimate tasks and perceived insider status. Thus, Hypothesis 3 is supported.

Model 3 involved a regression test of the control variables and illegitimate task on work withdrawal behavior. The results showed that the illegitimate task has a significant positive impact on work withdrawal behavior (Model 3, β = 0.316, *p* < 0.001). Hence, Hypothesis 1 is confirmed.

Model 4, building on Model 3, added perceived overqualification and the interaction term between perceived overqualification and the illegitimate task. The regression results showed that perceived overqualification positively moderates the relationship between the illegitimate task and work withdrawal behavior among the new generation (Model 4, β = 0.112, *p* < 0.05). Hence, Hypothesis 4 is supported.

**Figure 2:**
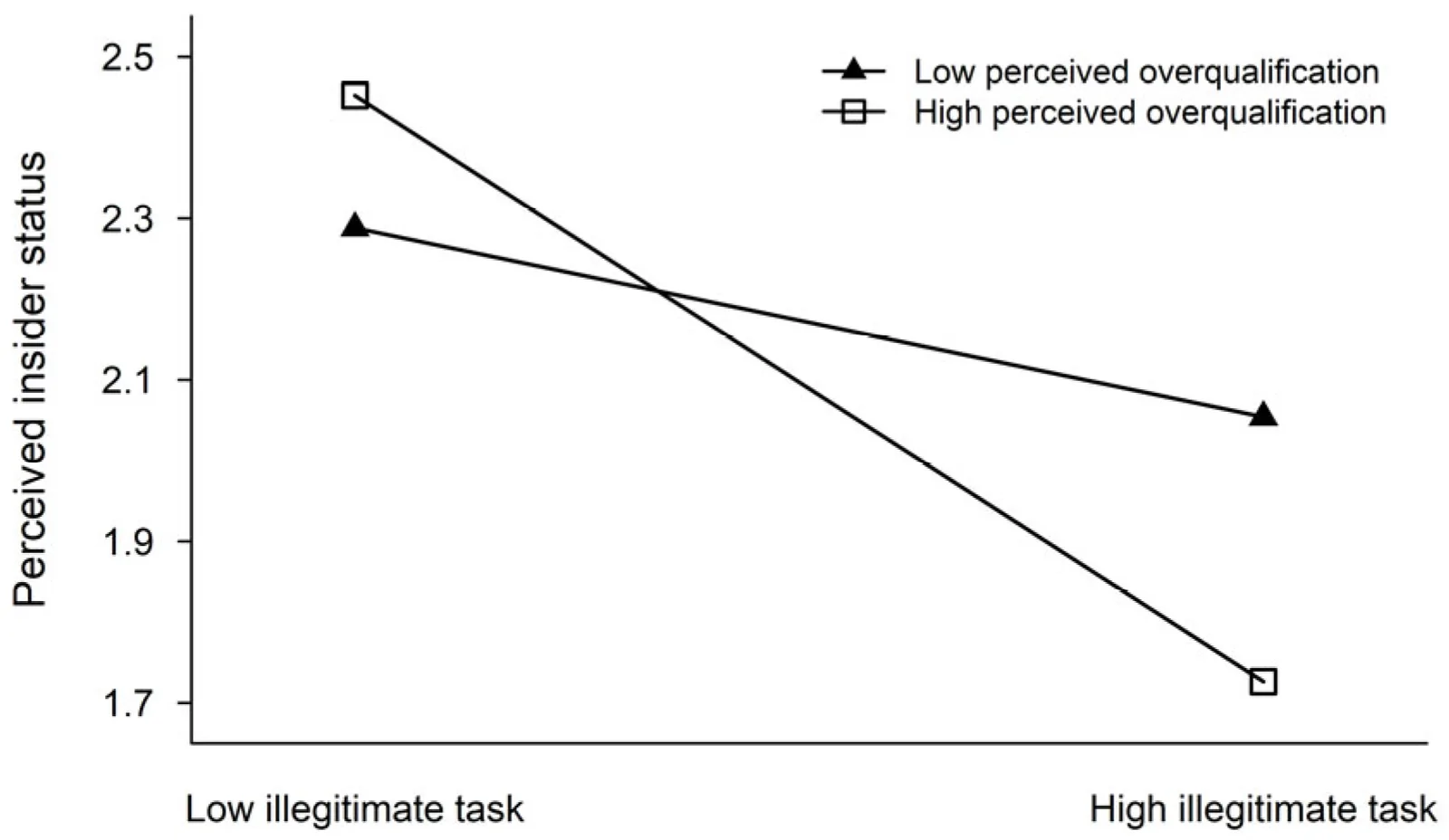
Diagram of the moderating effect of perceived overqualification (PIS).

**Figure 3:**
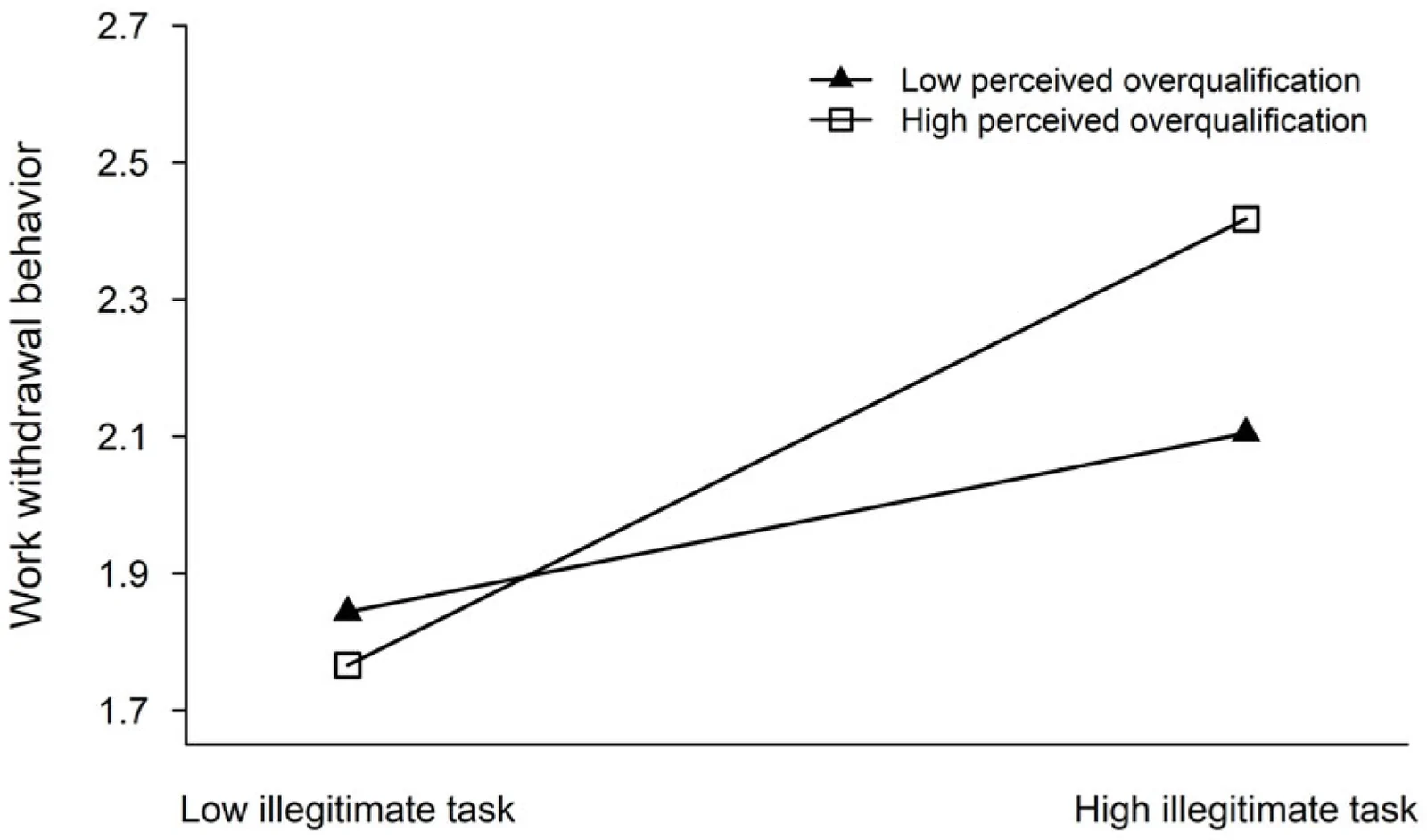
Diagram of the moderating effect of perceived overqualification (WWB).

To more intuitively display the moderating effect, we performed group regression fitting by adding and subtracting one standard deviation from the average perceived overqualification, forming simple slope graphs for low- and high-perceived overqualification. Figure 2 presents the simple slopes for the effect of illegitimate tasks on perceived insider status at low and high levels of perceived overqualification. The relationship is negative at both levels and is stronger among employees with high perceived overqualification than among those with low perceived overqualification. Figure 3 shows that the extent to which illegitimate tasks affect work withdrawal behavior is higher for employees with high-perceived overqualification than those with low-perceived overqualification.

#### 4.3.2. Mediating Effect Test

In Model 5, after including perceived insider status, illegitimate task remained positively associated with work withdrawal behavior (β = 0.180, *p* < 0.001), whereas perceived insider status was negatively associated with work withdrawal behavior (β = −0.378, *p* < 0.001). These results support the mediating role of perceived insider status [88]. The bootstrapped indirect effect remains positive because the two component paths are both negative (indirect = 0.133, 95% CI = [0.062, 0.222], *p* < 0.001; Table 4) [89]. Thus, Hypothesis 2 is confirmed. At the same time, the proportion of the indirect effect and the direct effect in the total effect was calculated, showing that the mediating effect accounted for 40.6% of the total effect, indicating a partial mediation effect [90].

#### 4.3.3. Moderated Mediation Test

Finally, we test the moderated mediation model. We distinguish between high- and low-score groups according to the mean of the moderating variable perceived overqualification, plus or minus one standard deviation. After 5000 bootstrap samples, we test the size and difference of the mediating effect in two groups of employees with different degrees of perceived overqualification [91]. By comparing whether there is a significant difference in the mediating effect between the two groups, we can determine whether the moderated mediation effect is established. As shown in Table 5, when perceived overqualification is high, the positive indirect effect of illegitimate tasks on employee work withdrawal behavior via perceived insider status is significant (β = 0.155, *p* < 0.05, 95% confidence interval [0.073, 0.252]). However, when perceived overqualification is low, the confidence interval for the indirect effect of illegitimate tasks on work withdrawal behavior via perceived insider status includes 0 (β = 0.054, *p* > 0.05, 95% confidence interval [−0.007, 0.129]). This shows that the mediation effect holds when perceived overqualification is high but does not hold when perceived overqualification is low, indicating the presence of a moderated mediation effect. Hypothesis 5 supports this.

**Figure 4:**
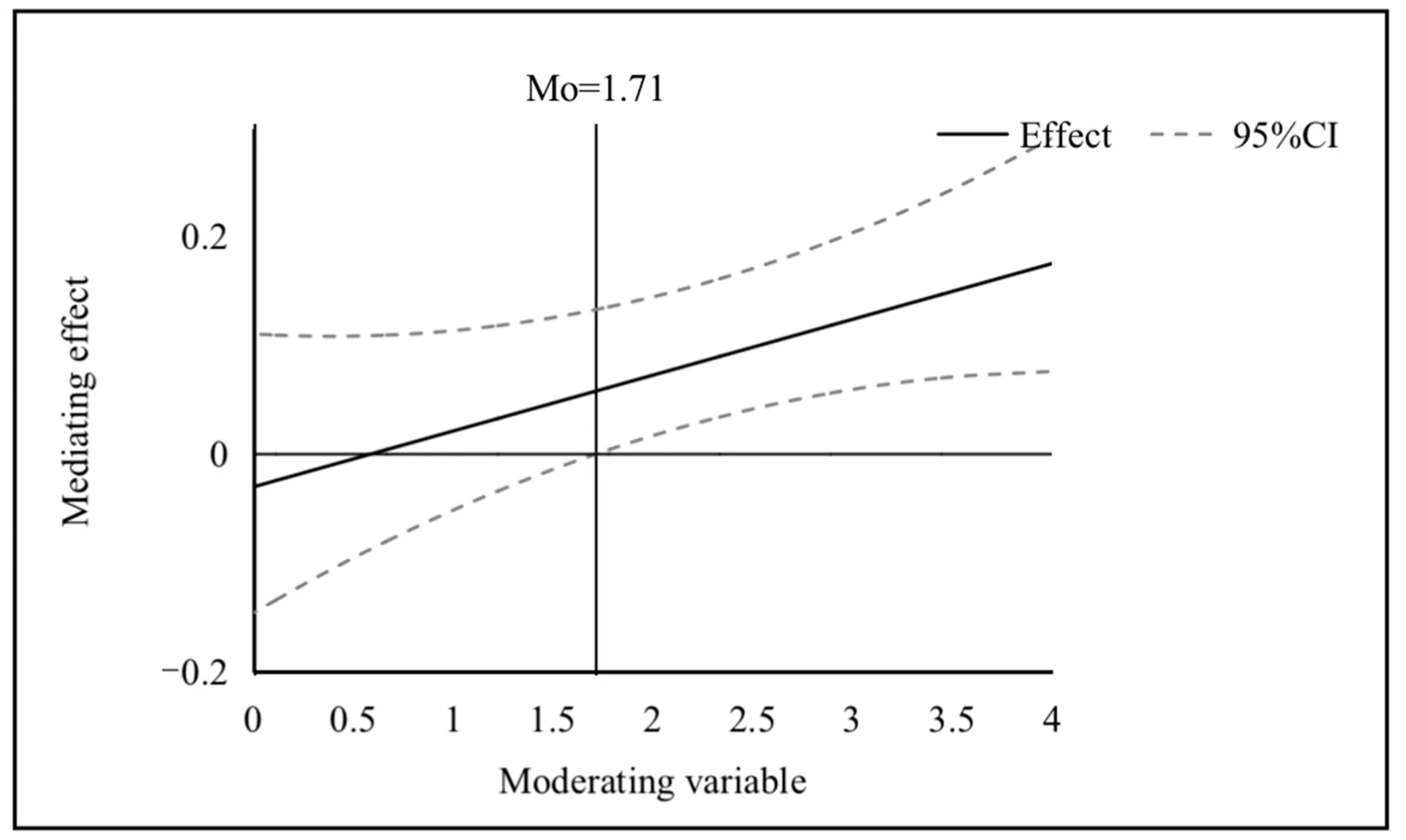
Diagram of regulatory intermediary effect.

As shown in the Figure 4, plotting the mediating effects and confidence intervals for different levels of regulation shows that the mediating effect holds when the moderating variable overqualification is greater than 1.71. When the excess qualification is less than 1.71, then the mediation effect does not hold.

## 5. Discussion

The main objective of this research is to broaden our understanding of why, how, and when illegitimate tasks may lead to work withdrawal behavior among Generation Z employees. We developed a theoretical model to determine how illegitimate tasks escalate work withdrawal behavior by damaging the perceived insider status. Additionally, we found that perceived overqualification enhances the impact of illegitimate tasks on perceived insider status and work withdrawal behavior. The effect of illegitimate tasks on the perceived insider status and work withdrawal behavior is more potent among highly perceived overqualified employees. The results of this research carry significant theoretical and practical implications.

### 5.1. Theoretical Contributions

Firstly, our research constructs the generation mechanism of work withdrawal behavior from the perspective of work tasks, expanding the understanding of the relationship between illegitimate tasks and work withdrawal behavior. Existing studies on intra-workplace precursors of withdrawal behavior concentrate on leaders, colleagues, and organizations [92,93,94], neglecting the element of work tasks. Our research indicates that illegitimate tasks lead employees to express dissatisfaction with the organization by engaging in withdrawal-related activities, revealing specific counterproductive behaviors in response to illegitimate tasks. In contrast, past research has only focused on general counterproductive behaviors. Our findings also suggest that Generation Z employees may choose to retaliate against unfair workplace treatment but will use covert behaviors.

Secondly, this research explores the mechanisms by which illegitimate tasks trigger withdrawal behavior from the perspective of perceived insider status. There are few studies in the literature related to the role of cognition when employees face illegitimate tasks. However, among the little knowledge of cognitive mechanisms, these studies have only focused on factors related to the job [95,96] and work [97,98], overlooking employees’ cognition towards the organization. Understanding whether cognition related to the organization will trigger work withdrawal behavior is very important for the organization to prevent such behavior from employees. Hence, this research expands the study of illegitimate tasks by revealing how such tasks can influence employees’ behavior through cognitive channels.

Thirdly, we propose and confirm that differences in perceived overqualification can explain employees’ reactions to illegitimate tasks. Perceived overqualification has received increasing attention in organizational behavior research due to its potential adverse effects on individuals and organizations [99]. However, previous studies have often used perceived overqualification as an antecedent variable. Our research confirms that overqualification, as a moderating variable, is key to understanding the complex relationship between illegitimate tasks and work withdrawal behavior. In other words, when employees feel that their qualifications exceed what their current jobs require, the impact of illegitimate tasks on their cognition and behaviors may be magnified. A significant proportion of Gen Z employees face the issue of overqualification in today’s workplace. Therefore, validating the negative role of overqualification as a boundary condition promotes organizations’ attention to and action on such issues among specific groups.

### 5.2. Practical Implications

Firstly, our research results indicate that illegitimate tasks can lead to work withdrawal behavior among Gen Z employees, who may seek to negatively affect the organization in covert ways. We encourage organizations to take effective intervention measures to minimize the presence of illegitimate tasks in the workplace. For instance, organizations should establish a system that clearly displays a tasks list for employees to ensure fair task distribution and reasonable work task boundaries. Organizations also need to train leaders in interpersonal interaction to reduce the sense of disrespect employees might feel to improve interaction fairness during task allocation. Moreover, leaders should provide detailed descriptions of work tasks to employees in a timely manner, helping them understand the rationality of tasks, which is crucial in reducing perceived illegitimate tasks.

Secondly, our research emphasizes the key role of perceived insider status. Fostering a sense of belonging and responsibility towards the organization among employees can help reduce their work withdrawal behavior. One suggestion is from the perspective of leaders, who can establish a harmonious interpersonal atmosphere, organize employee care activities, to foster a sense of belonging and identification among employees. Another suggestion is from the organizational perspective, which should establish a fair and caring corporate culture, enhance employees’ development and work experience, and let employees feel the care and respect of the organization.

Thirdly, organizations should pay attention to the problem of overqualification. High overqualification can amplify the negative effects of illegitimate tasks. To mitigate the negative impact of overqualification, organizations should pay attention to the job–person fit during the recruitment process. On the other hand, in view of the inevitable phenomenon of overqualification, leaders should provide regular psychological counseling services to employees to alleviate their sense of injustice based on overqualification.

Finally, managers should pay attention to the unique characteristics and behaviors of Gen Z employees. Managers should understand the generational differences in the workplace and how to deal with them. For example, managers should pay attention to the fairness of interaction and communicate and work with Gen Z employees on an equal footing. In addition, managers can close the psychological distance with Gen Z employees by participating in social media activities. These measures can effectively reduce management resistance.

### 5.3. Limitations and Directions for Future Research

While this study explores the impact of illegitimate tasks on the withdrawal behavior of Generation Z employees, offering relevant suggestions for the evolution and development of corporate management practices under modern conditions, certain limitations exist. First, this research investigates the influence of illegitimate tasks on the withdrawal behavior of Generation Z employees from the equity theory perspective. The equity theory alone may not fully capture the complexities of perceived illegitimate tasks and their relation to work withdrawal behavior. On the one hand, it cannot explain the multiple influences that coexist; on the other hand, it cannot reflect the impact of the interactions between various factors. Future studies could incorporate other theoretical perspectives, such as self-determination theory [82], for a more comprehensive investigation of the relationships between these variables. Secondly, the impact of illegitimate tasks on employee behavior and cognition involves diverse and complex mechanisms with various paths and theoretical models. Future research can explore the mechanisms by which illegitimate tasks influence withdrawal behavior from team or organizational perspectives [100]. Thirdly, the sample of this study comes from a single-age group, which limits the generalizability of the research conclusions to other age groups. In the future, we aim to gather a more diverse dataset, including employees of different ages, and focus on specific industries to gain more comprehensive insights. We will conduct a comparative analysis across different age groups. Meanwhile, considering that Gen Z employees have not fully entered the workforce, we plan to conduct the data analysis again at a future point in time. Finally, this study employs samples from Generation Z employees in Chinese organizations, meaning our results may not be generalizable to other countries. China’s culture of high power distance may affect the effects of illegitimate tasks. Future research can include non-Chinese samples to enhance our understanding of the mechanisms influencing illegitimate tasks.

## 6. Conclusions

This research focuses on the elaboration of the mechanisms of illegitimate tasks. Meanwhile, as Generation Z employees form a key part of organizations and significantly impact the sustainable development of organizations, they have been the primary focus of this study. Specifically, based on equity theory, this study finds that illegitimate tasks increase the withdrawal behavior of Generation Z employees by damaging their perceived insider status, providing a perspective on the relationship between the individual and the organization for future research on the mechanisms of illegitimate tasks. Simultaneously, this study identifies perceived overqualification as a boundary condition for this mechanism, providing a new direction for research on perceived overqualification.

## Figures and Tables

**Table 1:** Confirmatory factor analysis.

Model	Factor	$\chi^{2}$	$\chi^{2}/df$	CFI	TLI	RMSEA	SRMR
Model 1	Four factors + potential methodological factors	1152.088	1.979	0.932	0.925	0.066	0.045
Model 2	Four factors (IT, PIS, POQ, WWB)	1370.107	2.199	0.930	0.926	0.067	0.048
Model 3	Three factors (IT + PIS, POQ, WWB)	4116.283	17.098	0.674	0.654	0.144	0.180
Model 4	Three factors (IT, PIS + POQ, WWB)	4113.999	6.572	0.675	0.654	0.144	0.179
Model 5	Two factors (IT + PIS + POQ WWB)	5161.164	8.218	0.577	0.552	0.164	0.202
Model 6	Single factor (IT +PIS + POQ + WWB)	6763.918	10.753	0.428	0.394	0.190	0.229

Note: IT = Illegitimate task, PIS = Perceived insider status, POQ = Perceived overqualification, WWB = Work withdrawal behavior.

**Table 2:** Correlation analysis results.

Variables	Mean	SD	1	2	3	4	5	6	7	8	9
1. Gender	0.43	0.50	1								
2. Age	24.91	2.06	−0.022	1							
3. Education	1.94	0.44	0.076	−0.054	1						
4. Working years	2.87	0.90	−0.05	−0.104	−0.140 *	1					
5. Industry	1.56	1.15	0.018	0.069	0.040	−0.048	1				
6. Enterprise category	1.79	0.70	−0.037	0.046	−0.074	−0.02	0.051	1			
7. Illegitimate task	2.55	0.83	−0.063	0.086	−0.074	−0.06	0.140 *	0.052	1		
8. Perceived overqualification	2.64	1.05	−0.016	0.055	−0.001	0.032	−0.004	−0.057	0.055	1	
9. Perceived insider status	2.14	0.83	−0.134 *	0.058	−0.021	−0.162 **	−0.002	0.06	−0.330 **	−0.086	1
10. Work withdrawal behavior	2.04	0.83	−0.036	0.052	−0.039	−0.214 **	0.088	0.146 *	0.325 **	0.092	−0.463 **

Note: ** *p* < 0.01; * *p* < 0.05.

**Table 3:** Hierarchical regression analysis results.

Variable	Perceived Insider Status		Work Withdrawal Behavior		
	M1	M2	M3	M4	M5
Gender	−0.196 * (0.096)	−0.185 (0.094)	−0.028 (0.095)	−0.023 (0.094)	0.041 (0.089)
Education	−0.007 (0.109)	−0.037 (0.108)	−0.039 (0.109)	−0.092 (0.108)	−0.043 (0.101)
Age	0.006 (0.098)	0.001 (0.097)	−0.213 * (0.097)	−0.213 * (0.096)	−0.134 (0.091)
Working years	−0.139 (0.065)	−0.151 ** (0.064)	−0.102 (0.065)	−0.195 *** (0.064)	0.131 ** (0.060)
Industry	0.045 (0.042)	−0.047 (0.041)	0.023 (0.042)	0.018 (0.041)	0.038 (0.039)
Enterprise category	0.045 (0.067)	0.067 (0.066)	0.140 (0.067)	0.165 * (0.066)	0.128 * (0.062)
Illegitimate task	−0.322 *** (0.058)	−0.289 *** (0.057)	0.316 *** (0.057)	0.275 *** (0.057)	0.180 *** (0.056)
Perceived insider status	-	-	-	-	−0.378 *** (0.057)
Perceived overqualification	-	−0.039 (0.045)	-	0.056 (0.045)	-
Illegitimate task × Perceived overqualification	-	−0.141 ** (0.044)	-	0.112 * (0.044)	-
Constant	2.137 *** (0.047)	2.130 *** (0.047)	2.038 *** (0.047)	2.033 *** (0.047)	2.038 *** (0.043)
F	7.023 ***	10.030 ***	7.615 ***	6.390 ***	14.610 ***
Adjusted R^2^	0.148	0.157	0.140	0.162	0.262

Note: *** *p* < 0.001; ** *p* < 0.01; * *p* < 0.05; values in parentheses are standard errors.

**Table 4:** Bootstrap mediating effect Test Results.

Effect Value	Estimate	SE	95% Confidence Interval		Z	p	% Mediation
Indirect effects	0.133	0.0398	0.062	0.222	3.33	<0.001	40.6
Direct effects	0.194	0.0584	0.084	0.311	3.33	<0.001	59.4
Total effects	0.327	0.0661	0.201	0.463	4.95	<0.001	100.0

**Table 5:** Results of bootstrap moderation estimates.

Path	Estimate	Boot SE	z	*p*	95% Confidence Interval	
					Lower	Upper
Low (−1 SD)	0.054	0.034	1.592	0.111	−0.007	0.129
Average	0.105	0.033	3.200	0.001 **	0.046	0.175
High (+1 SD)	0.155	0.045	3.457	<0.001 ***	0.073	0.252

Note: ** *p* < 0.01; *** *p* < 0.001; values in parentheses are standard errors.

## Data Availability

The data used to support the findings of this study are included within the article.

## Appendix A

1. Scale of Illegitimate Task

The following statements relate to your Illegitimate Task. Please indicate how frequently each statement applies to you. (1 = never, 2 = rarely, 3 = sometimes, 4 = often, 5 = always).

**Table A1:** Scale of Illegitimate Task.

Item	Never	Rarely	Sometimes	Often	Always
1. I have work tasks to take care of, which keep me wondering if they even have to be done at all	1	2	3	4	5
2. I get tasks I don’t see any point in doing	1	2	3	4	5
3. I have work tasks to attend to, which seem a waste of time	1	2	3	4	5
4. I get work tasks that, in my opinion, should be done by someone else	1	2	3	4	5
5. I receive tasks that can be problematic considering my core job duties	1	2	3	4	5
6. I receive tasks in my work that do not correspond with my role	1	2	3	4	5
7. I am expected to do things in my work that seem unnecessary in view of my actual work assignments	1	2	3	4	5
8. I am given work tasks without adequate resources to complete them	1	2	3	4	5

2. Scale of Perceived Overqualification

The following statements relate to your Perceived Overqualification. Please indicate how frequently each statement applies to you. (1 = never, 2 = rarely, 3 = sometimes, 4 = often, 5 = always).

**Table A2:** Scale of Perceived Overqualification.

Item	Never	Rarely	Sometimes	Often	Always
1. My job requires less education than I have	1	2	3	4	5
2. The work experience that I have is not necessary to be successful on this job	1	2	3	4	5
3. I have job skills that are not required for this job	1	2	3	4	5
4. Someone with less education than myself could perform well on my job	1	2	3	4	5
5. My previous training is not being fully utilized on this job	1	2	3	4	5
6. I have a lot of knowledge that I do not need in order to do my job	1	2	3	4	5
7. My education level is above the education level required by my jo	1	2	3	4	5
8. Someone with less work experience than myself could do my job just as well	1	2	3	4	5
9. I have more abilities than I need in order to do my job	1	2	3	4	5

3. Scale of Perceived Insider Status

The following statements relate to your Perceived Insider Status. Please indicate how frequently each statement applies to you. (1 = never, 2 = rarely, 3 = sometimes, 4 = often, 5 = always).

**Table A3:** Scale of Perceived Insider Status.

Item	Never	Rarely	Sometimes	Often	Always
1. I feel very much a part of my work organization	1	2	3	4	5
2. My work organization makes me believe that I am included in it	1	2	3	4	5
3. I feel like I am an ‘outsider’ at this organization	1	2	3	4	5
4. I don’t feel included in this organization	1	2	3	4	5
5. I feel I am an ‘insider’ in my work organization	1	2	3	4	5
6. My work organization makes me frequently feel ‘left-out’	1	2	3	4	5

4. Scale of Work Withdrawal Behavior

The following statements relate to your Work Withdrawal Behavior. Please indicate how frequently each statement applies to you. (1 = never, 2 = rarely, 3 = sometimes, 4 = often, 5 = always).

**Table A4:** Scale of Perceived Insider Status.

Item	Never	Rarely	Sometimes	Often	Always
1. Thoughts of being absent	1	2	3	4	5
2. Chat with co-workers about nonwork topics	1	2	3	4	5
3. Left work station for unnecessary reasons	1	2	3	4	5
4. Daydreaming	1	2	3	4	5
5. Spent work time on personal matters	1	2	3	4	5
6. Put less effort into job than should have	1	2	3	4	5
7. Thoughts of leaving current job	1	2	3	4	5
8. Let others do your work	1	2	3	4	5
9. Left work early without permission	1	2	3	4	5
10. Taken longer lunch or rest break than allowed	1	2	3	4	5
11. Taken supplies or equipment without permission	1	2	3	4	5
12. Fallen asleep at work	1	2	3	4	5
