# Correction: Fan et al. (2023). Do Illegitimate Tasks Lead to Work Withdrawal Behavior Among Generation Z Employees in China? The Role of Perceived Insider Status and Overqualification. *Behavioral Sciences*, *13*(9), 702

**DOI:** 10.3390/bs16081407

**Published:** 2026-08-17

**Authors:** Pengxiang Fan, Hao Zhang, Songlin Yang, Zixuan Yu, Ming Guo

**Affiliations:** 1 School of Economics and Management, Beijing Jiaotong University, Beijing 100089, China; 2 School of Economics, University of Amsterdam, 1018 WV Amsterdam, The Netherlands

Background and Scope of the Correction

In the original publication (Fan et al., 2023), several minus signs were inadvertently omitted when statistical outputs were transferred into the manuscript tables and associated text. To determine the full scope of the problem, the authors rechecked the original data, variable-scoring procedures, correlation analyses, regression analyses, and moderated-mediation tests. An independent cross-check was conducted by individuals who had not participated in the original data analysis.

The reanalysis of the 283 valid observations confirmed that, apart from minor differences in the final decimal place arising from rounding, the absolute values of the affected correlation and regression coefficients were consistent with those reported in the published article. The corrections primarily concern the signs of coefficients involving perceived insider status, the corresponding simple-slope plot, and the related descriptions of the directions of these statistical relationships in the main text.

Error in Table 2

In the original publication, there were mistakes in Table 2 as published. Minus signs were omitted for the correlations between illegitimate task and perceived insider status (published: 0.330; corrected: −0.330), perceived overqualification and perceived insider status (published: 0.086; corrected: −0.086), and perceived insider status and work withdrawal behavior (published: 0.463; corrected: −0.463). The corrected Table 2 appears below.

**Table 2:** Correlation analysis results.

Variables	Mean	SD	1	2	3	4	5	6	7	8	9
1. Gender	0.43	0.50	1								
2. Age	24.91	2.06	−0.022	1							
3. Education	1.94	0.44	0.076	−0.054	1						
4. Working years	2.87	0.90	−0.05	−0.104	−0.140 *	1					
5. Industry	1.56	1.15	0.018	0.069	0.040	−0.048	1				
6. Enterprise category	1.79	0.70	−0.037	0.046	−0.074	−0.02	0.051	1			
7. Illegitimate task	2.55	0.83	−0.063	0.086	−0.074	−0.06	0.140 *	0.052	1		
8. Perceived overqualification	2.64	1.05	−0.016	0.055	−0.001	0.032	−0.004	−0.057	0.055	1	
9. Perceived insider status	2.14	0.83	−0.134 *	0.058	−0.021	−0.162 **	−0.002	0.06	−0.330 **	−0.086	1
10. Work withdrawal behavior	2.04	0.83	−0.036	0.052	−0.039	−0.214 **	0.088	0.146 *	0.325 **	0.092	−0.463 **

Note: ** *p* < 0.01; * *p* < 0.05.

Error in Table 3

In the original publication, there were mistakes in Table 3 as published. Minus signs were omitted for the coefficients of illegitimate task on perceived insider status in M1 (published: 0.320; corrected: −0.322), illegitimate task in M2 (published: 0.290; corrected: −0.289), perceived overqualification in M2 (published: 0.039; corrected: −0.039), the interaction between illegitimate task and perceived overqualification in M2 (published: 0.141; corrected: −0.141), and perceived insider status on work withdrawal behavior in M5 (published: 0.378; corrected: −0.378).

**Table 3:** Hierarchical regression analysis results.

Variable	Perceived Insider Status		Work Withdrawal Behavior		
	M1	M2	M3	M4	M5
Gender	−0.196 * (0.096)	−0.185 (0.094)	−0.028 (0.095)	−0.023 (0.094)	0.041 (0.089)
Education	−0.007 (0.109)	−0.037 (0.108)	−0.039 (0.109)	−0.092 (0.108)	−0.043 (0.101)
Age	0.006 (0.098)	0.001 (0.097)	−0.213 * (0.097)	−0.213 * (0.096)	−0.134 (0.091)
Working years	−0.139 (0.065)	−0.151 ** (0.064)	−0.102 (0.065)	−0.195 *** (0.064)	0.131 ** (0.060)
Industry	0.045 (0.042)	−0.047 (0.041)	0.023 (0.042)	0.018 (0.041)	0.038 (0.039)
Enterprise category	0.045 (0.067)	0.067 (0.066)	0.140 (0.067)	0.165 * (0.066)	0.128 * (0.062)
Illegitimate task	−0.322 *** (0.058)	−0.289 *** (0.057)	0.316 *** (0.057)	0.275 *** (0.057)	0.180 *** (0.056)
Perceived insider status	-	-	-	-	−0.378 *** (0.057)
Perceived overqualification	-	−0.039 (0.045)	-	0.056 (0.045)	-
Illegitimate task × Perceived overqualification	-	−0.141 ** (0.044)	-	0.112 * (0.044)	-
Constant	2.137 *** (0.047)	2.130 *** (0.047)	2.038 *** (0.047)	2.033 *** (0.047)	2.038 *** (0.043)
F	7.023 ***	10.030 ***	7.615 ***	6.390 ***	14.610 ***
Adjusted R^2^	0.148	0.157	0.140	0.162	0.262

Note: *** *p* < 0.001; ** *p* < 0.01; * *p* < 0.05; values in parentheses are standard errors.

Error in Figure 2 and Formatting Update to Figure 3

Because the signs of the Model 2 coefficients involving perceived insider status were reported incorrectly, the published Figure 2 displayed the simple slopes in the wrong direction. The corrected Figure 2 shows that illegitimate tasks are negatively associated with perceived insider status and that this negative relationship is stronger when perceived overqualification is high. Figure 3 has also been updated solely to ensure consistency in formatting and presentation with the corrected Figure 2. This formatting update does not involve any change to the statistical results or their interpretation. The corrected Figure 2 and reformatted Figure 3 appear below.

**Figure 2:**
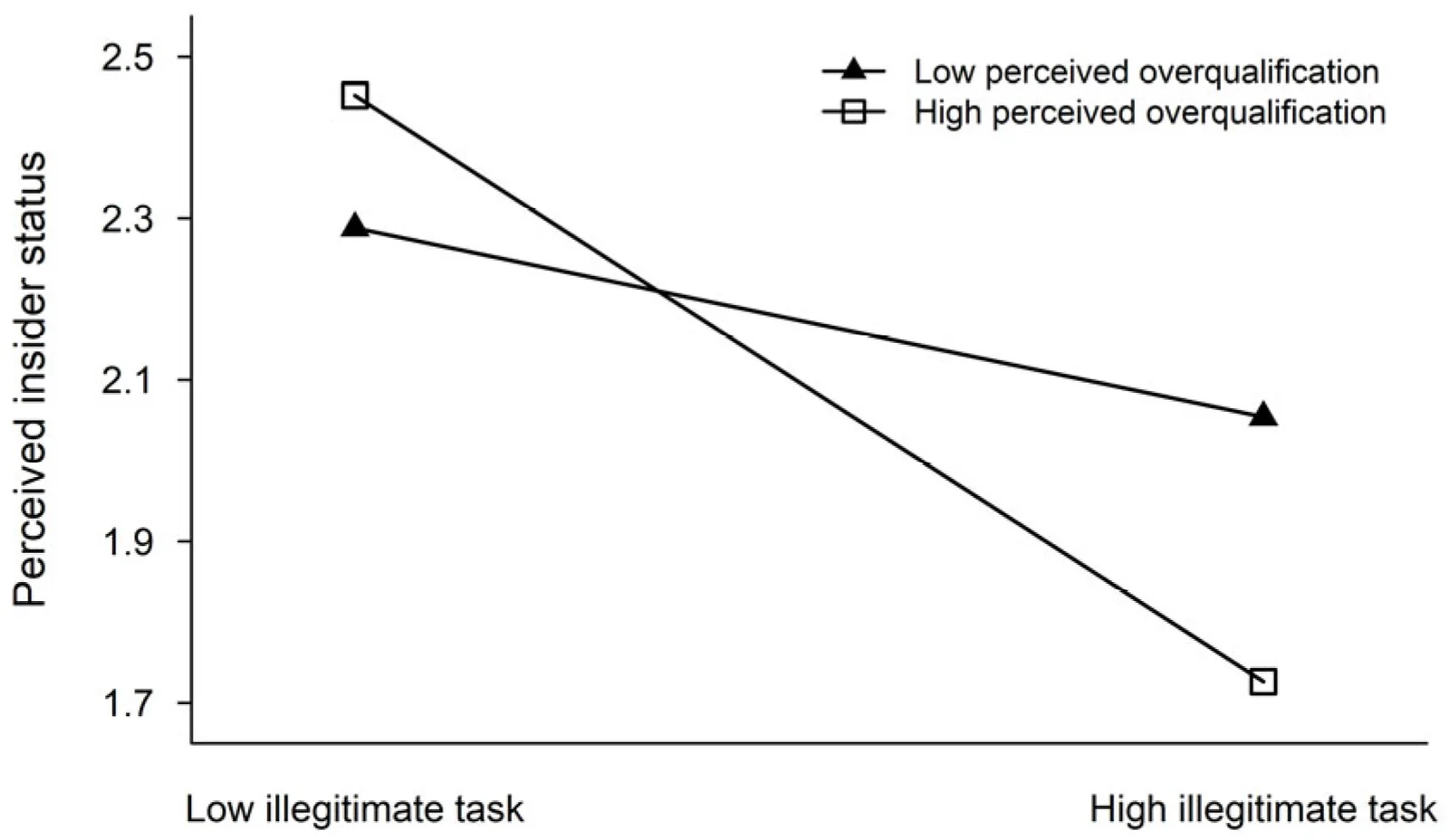
Diagram of the moderating effect of perceived overqualification (PIS).

**Figure 3:**
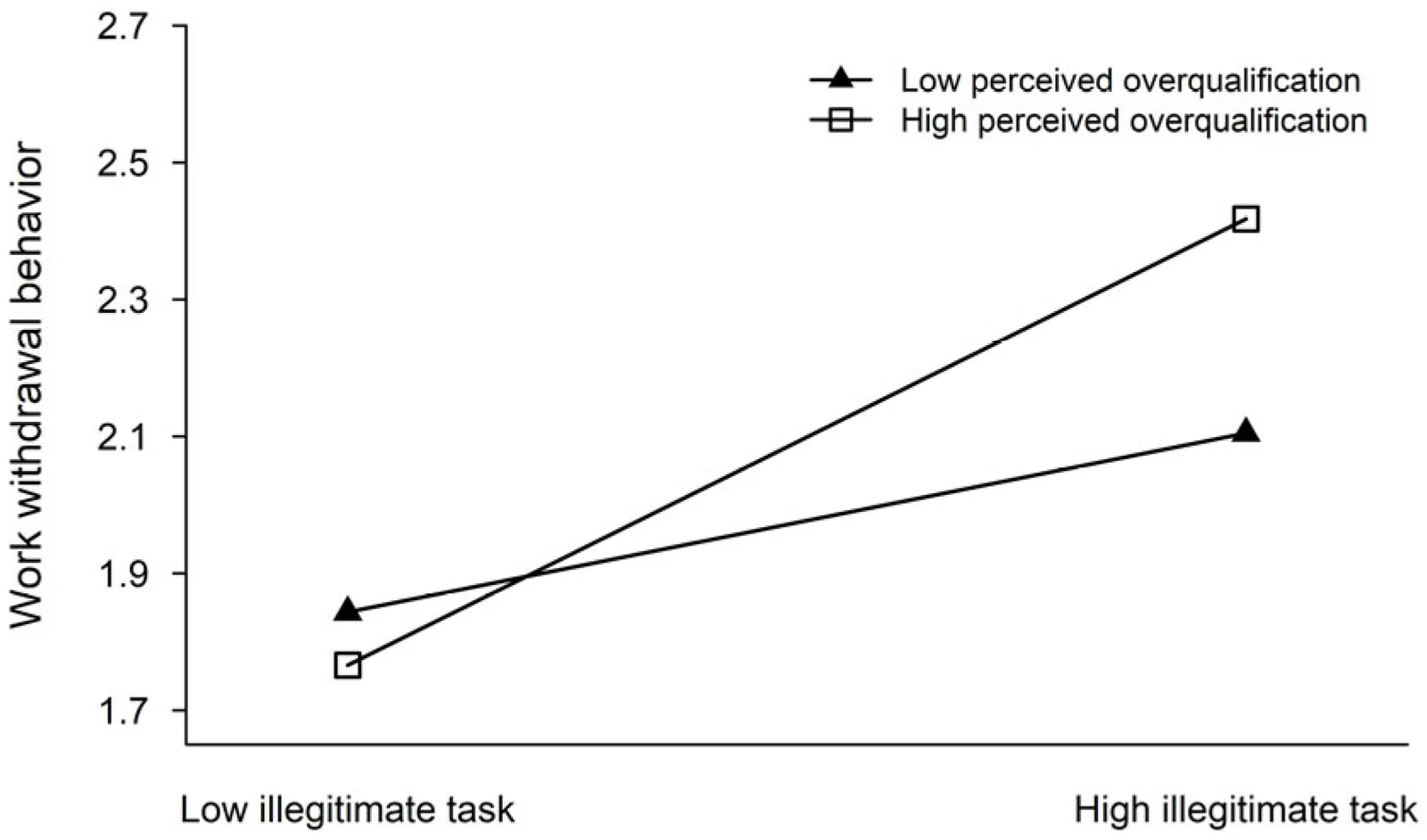
Diagram of the moderating effect of perceived overqualification (WWB).

Text Correction

The coefficient-sign errors also affected several sentences in the main text. The corrected wording is provided below. Unaffected sentences and paragraphs remain unchanged.

Section 4.2, Descriptive Statistical Analysis

The first paragraph should read:

“Table 2 presents the means, standard deviations, and correlation coefficients of the study variables. The results show that illegitimate tasks were significantly and negatively correlated with perceived insider status (r = −0.330, *p* < 0.01) and significantly and positively correlated with work withdrawal behavior (r = 0.325, *p* < 0.01). Perceived insider status was significantly and negatively correlated with work withdrawal behavior (r = −0.463, *p* < 0.01). These correlations align with the theoretical model and provide preliminary support for the hypothesis testing.”

Section 4.3.1, Main Effect and Moderating Effect Tests

The paragraph describing Model 1 should read:

“In Model 1, only the control variables and illegitimate task were included in the regression. The results showed a significant negative association between illegitimate task and perceived insider status (Model 1, β = −0.322, *p* < 0.001).”

The paragraph describing Model 2 should read:

“Model 2, based on Model 1, added perceived overqualification and the interaction term between illegitimate task and perceived overqualification. Illegitimate task remained negatively associated with perceived insider status (β = −0.289, *p* < 0.001). The interaction between illegitimate task and perceived overqualification was negative and significant (β = −0.141, *p* < 0.01), indicating that perceived overqualification strengthens the negative relationship between illegitimate tasks and perceived insider status. Thus, Hypothesis 3 is supported.”

The sentences interpreting Figure 2 should read:

“Figure 2 presents the simple slopes for the effect of illegitimate tasks on perceived insider status at low and high levels of perceived overqualification. The relationship is negative at both levels and is stronger among employees with high perceived overqualification than among those with low perceived overqualification.”

Section 4.3.2, Mediating Effect Test

The opening sentences should read:

“In Model 5, after including perceived insider status, illegitimate task remained positively associated with work withdrawal behavior (β = 0.180, *p* < 0.001), whereas perceived insider status was negatively associated with work withdrawal behavior (β = −0.378, *p* < 0.001). These results support the mediating role of perceived insider status [88]. The bootstrapped indirect effect remains positive because the two component paths are both negative (indirect = 0.133, 95% CI = [0.062, 0.222], *p* < 0.001; Table 4) [89]. Thus, Hypothesis 2 is confirmed.”

Conclusions

The corrected mechanism is that illegitimate tasks reduce employees’ perceived insider status, while perceived insider status reduces work withdrawal behavior. Because both component paths of the indirect effect are negative, their product remains positive. Therefore, the direction and magnitude of the indirect effect (0.133), the core mediation conclusion, and the moderated-mediation conclusion remain unchanged. The interaction effect associated with Hypothesis 4 (0.112) is also unchanged. Figure 3 has been updated solely for consistency in formatting and presentation; no changes were made to its statistical results or interpretation. No corrections are required to Table 4 or Table 5.

The authors apologize for the errors and accept collective responsibility for the published version. The authors state that these corrections do not affect the scientific conclusions. This correction is submitted for approval by the Academic Editor; upon approval, the original publication should be updated accordingly.
